# Methodological considerations in injury burden of disease studies across Europe: a systematic literature review

**DOI:** 10.1186/s12889-022-13925-z

**Published:** 2022-08-17

**Authors:** Periklis Charalampous, Elena Pallari, Vanessa Gorasso, Elena von der Lippe, Brecht Devleesschauwer, Sara M. Pires, Dietrich Plass, Jane Idavain, Che Henry Ngwa, Isabel Noguer, Alicia Padron-Monedero, Rodrigo Sarmiento, Marek Majdan, Balázs Ádám, Ala’a AlKerwi, Seila Cilovic-Lagarija, Benjamin Clarsen, Barbara Corso, Sarah Cuschieri, Keren Dopelt, Mary Economou, Florian Fischer, Alberto Freitas, Juan Manuel García-González, Federica Gazzelloni, Artemis Gkitakou, Hakan Gulmez, Paul Hynds, Gaetano Isola, Lea S. Jakobsen, Zubair Kabir, Katarzyna Kissimova-Skarbek, Ann Kristin Knudsen, Naime Meriç Konar, Carina Ladeira, Brian Lassen, Aaron Liew, Marjeta Majer, Enkeleint A. Mechili, Alibek Mereke, Lorenzo Monasta, Stefania Mondello, Joana Nazaré Morgado, Evangelia Nena, Edmond S. W. Ng, Vikram Niranjan, Iskra Alexandra Nola, Rónán O’Caoimh, Panagiotis Petrou, Vera Pinheiro, Miguel Reina Ortiz, Silvia Riva, Hanen Samouda, João Vasco Santos, Cornelia Melinda Adi Santoso, Milena Santric Milicevic, Dimitrios Skempes, Ana Catarina Sousa, Niko Speybroeck, Fimka Tozija, Brigid Unim, Hilal Bektaş Uysal, Fabrizio Giovanni Vaccaro, Orsolya Varga, Milena Vasic, Francesco Saverio Violante, Grant M. A. Wyper, Suzanne Polinder, Juanita A. Haagsma

**Affiliations:** 1 Department of Public Health, Erasmus MC University Medical Center, Rotterdam, The Netherlands; 2 Health Innovation Network, Minerva House, Montague Close, London, UK; 3 Department of Public Health and Primary Care, Ghent University, Ghent, Belgium; 4 Department of Epidemiology and Public Health, Sciensano, Brussels, Belgium; 5 Department of Epidemiology and Health Monitoring, Robert Koch Institute, Berlin, Germany; 6 Department of Translational Physiology, Infectiology and Public Health, Ghent University, Merelbeke, Belgium; 7 National Food Institute, Technical University of Denmark, Lyngby, Denmark; 8 Department for Exposure Assessment, and Environmental Health Indicators, German Environment Agency, Berlin, Germany; 9 Department of Health Statistics, National Institute for Health Development, Tallinn, Estonia; 10 School of Public Health and Community Medicine, Sahlgrenska Academy, University of Gothenburg, Gothenburg, Sweden; 11 Department of Epidemiology and Population Health, Faculty of Health Sciences, American University of Beirut, Beirut, Lebanon; 12 National School of Public Health, Carlos III Institute of Health, Madrid, Spain; 13 Medicine School, University of Applied and Environmental Sciences, Bogota, Colombia; 14 Department of Public Health, Faculty of Health Sciences and Social Work, Trnava University, Trnava, Slovakia; 15 Institute of Public Health, College of Medicine and Health Sciences, United Arab Emirates University, Al Ain, United Arab Emirates; 16 Department of Public Health and Epidemiology, Faculty of Medicine, University of Debrecen, Debrecen, Hungary; 17 Directorate of Health, Service Epidemiology and Statistics, Luxembourg, Luxembourg; 18 Institute for Public Health FB&H, Sarajevo, Sarajevo, Bosnia and Herzegovina; 19 Sports Trauma Research Center, Department of Sports Medicine, Norwegian School of Sport Sciences, Oslo, Norway; 20 Department of Disease Burden, Norwegian Institute of Public Health, Bergen, Norway; 21 Department of Health and Functioning, Faculty of Health and Social Sciences, Western Norway University of Applied Sciences, Bergen, Norway; 22 Institute of Neuroscience, National Research Council, Rome, Italy; 23 Department of Anatomy, Faculty of Medicine and Surgery, University of Malta, Msida, Malta; 24 Department of Public Health, Ashkelon Academic College, Ashkelon, Israel; 25 Department of Health Policy and Management, School of Public Health, Faculty of Health Sciences, Ben-Gurion University of the Negev, Beer Sheva, Israel; 26 Department of Nursing, School of Health Sciences, Cyprus University of Technology, Limassol, Cyprus; 27 Institute of Public Health, Charité - Universitätsmedizin Berlin, Berlin, Germany; 28 CINTESIS – Center for Health Technology and Services Research, Faculty of Medicine, University of Porto, Porto, Portugal; 29 Department of Community Medicine, Information and Health Decision Sciences (MEDCIDS), Faculty of Medicine, University of Porto, Porto, Portugal; 30 Department of Sociology, Universidad Pablo de Olavide, Seville, Spain; 31 Institute and Faculty of Actuaries, London, UK; 32 Department of Internal Medicine and Epidemiology, Erasmus MC University Medical Center, Rotterdam, The Netherlands; 33 Department of Family Medicine, Faculty of Medicine, İzmir Democracy University, Izmir, Turkey; 34 Environmental Sustainability and Health Institute, Technological University Dublin, Dublin, Ireland; 35 Department of General Surgery and Surgical-Medical Specialties, School of Dentistry, University of Catania, Catania, Italy; 36 Public Health & Epidemiology, School of Public Health, University College Cork, Cork, Ireland; 37 Department of Health Economics and Social Security, Faculty of Health Sciences, Jagiellonian University Medical College, Krakow, Poland; 38 Department of Biostatistics and Medical Informatics, Faculty of Medicine, Kirsehir Ahi Evran University, Kirsehir, Turkey; 39 H&TRC – Health & Technology Research Center, Escola Superior de Tecnologia da Saúde (ESTeSL), Instituto Politécnico de Lisboa, Lisbon, Portugal; 40 Comprehensive Health Research Centre (CHRC), Universidade NOVA de Lisboa, Lisbon, Portugal; 41 Clinical Sciences Institute, School of Medicine, National University of Ireland, Galway, Galway City, Ireland; 42 Andrija Štampar School of Public Health, School of Medicine, University of Zagreb, Zagreb, Croatia; 43 Clinic of Social and Family Medicine, School of Medicine, University of Crete, Crete, Greece; 44 Department of Healthcare, Faculty of Public Health, University of Vlora, Vlora, Albania; 45 Al-Farabi Kazakh National University, Almaty, Kazakhstan; 46 Institute of Maternal, Child Health - IRCCS Burlo Garofolo, Trieste, Italy; 47 Department of Biomedical and Dental Sciences and Morphofunctional Imaging, University of Messina, Messina, Italy; 48 Environmental Health and Nutrition Laboratory, Faculty of Medicine, University of Lisbon, Lisbon, Portugal; 49 Laboratory of Social Medicine, Medical School, Democritus University of Thrace, Alexandroupolis, Greece; 50 School of Hygiene & Tropical Medicine, London, UK; 51 School of Public Health, Physiotherapy and Sport Sciences, University College Dublin, Dublin, Ireland; 52 Department of Geriatric Medicine, Mercy University Hospital, Grenville Place, Cork City, Ireland; 53 Pharmacoepidemiology-Pharmacovigilance, Pharmacy School, School of Sciences and Engineering, University of Nicosia, Nicosia, Cyprus; 54 College of Public Health, University of South Florida, Tampa, FL USA; 55 Department of Psychology and Pedagogic Science, St Mary’s University, London, UK; 56 Population Health Department, Luxembourg Institute of Health, Nutrition and Health Research Group, Luxembourg, Luxembourg; 57 Public Health Unit, ACES Grande Porto VIII - Espinho/Gaia, ARS Norte, Lisbon, Portugal; 58 Faculty of Public Health, University of Debrecen, Debrecen, Hungary; 59 Institute of Social Medicine, Faculty of Medicine, University of Belgrade, Belgrade, Serbia; 60 Swiss Paraplegic Research, Nottwil, Switzerland; 61 Department of Biology, School of Science and Technology, University of Évora, Évora, Portugal; 62 Comprehensive Health Research Centre (CHRC), University of Évora, Évora, Portugal; 63 Institute of Health and Society (IRSS), Université Catholique de Louvain, Brussels, Belgium; 64 Institute of Public Health of Republic of North Macedonia, Saints Cyril and Methodius University of Skopje, Skopje, North Macedonia; 65 Faculty of Medicine, Saints Cyril and Methodius University of Skopje, Skopje, North Macedonia; 66 Department of Cardiovascular, Endocrine-Metabolic Diseases and Aging, Istituto Superiore Di Sanità, Rome, Italy; 67 Department of Internal Medicine, Adnan Menderes University School of Medicine, Aydin, Turkey; 68 School of Public Health, Università Vita-Salute San Raffaele, Milan, Italy; 69 Faculty of Dentistry Pancevo, University Business Academy in Novi Sad, Pancevo, Serbia; 70 Institute of Public Health of Serbia Dr Milan Jovanović Batut, Belgrade, Serbia; 71 Department of Medical and Surgical Sciences, Alma Mater Studiorum University of Bologna, Bologna, Italy; 72 Unit of Occupational Medicine, IRCCS Azienda Ospedaliero-Universitaria Di Bologna, Bologna, Italy; 73 Place and Wellbeing Directorate, Public Health Scotland, Glasgow, Scotland, UK

**Keywords:** Burden of disease, Burden of Injury, Disability-adjusted life years, Review, Methodology

## Abstract

**Background:**

Calculating the disease burden due to injury is complex, as it requires many methodological choices. Until now, an overview of the methodological design choices that have been made in burden of disease (BoD) studies in injury populations is not available. The aim of this systematic literature review was to identify existing injury BoD studies undertaken across Europe and to comprehensively review the methodological design choices and assumption parameters that have been made to calculate years of life lost (YLL) and years lived with disability (YLD) in these studies.

**Methods:**

We searched EMBASE, MEDLINE, Cochrane Central, Google Scholar, and Web of Science, and the grey literature supplemented by handsearching, for BoD studies. We included injury BoD studies that quantified the BoD expressed in YLL, YLD, and disability-adjusted life years (DALY) in countries within the European Region between early-1990 and mid-2021.

**Results:**

We retrieved 2,914 results of which 48 performed an injury-specific BoD assessment. Single-country independent and Global Burden of Disease (GBD)-linked injury BoD studies were performed in 11 European countries. Approximately 79% of injury BoD studies reported the BoD by external cause-of-injury. Most independent studies used the incidence-based approach to calculate YLDs. About half of the injury disease burden studies applied disability weights (DWs) developed by the GBD study. Almost all independent injury studies have determined YLL using national life tables.

**Conclusions:**

Considerable methodological variation across independent injury BoD assessments was observed; differences were mainly apparent in the design choices and assumption parameters towards injury YLD calculations, implementation of DWs, and the choice of life table for YLL calculations. Development and use of guidelines for performing and reporting of injury BoD studies is crucial to enhance transparency and comparability of injury BoD estimates across Europe and beyond.

**Supplementary Information:**

The online version contains supplementary material available at 10.1186/s12889-022-13925-z.

## Background

Across the global burden of disease (BoD) landscape, injuries are a major public health problem. There have been significant declines in case fatality rates from severe injury over recent decades, indicating that access to trauma care systems have led to improvements in survival [1, 2]. However, survivors of severe injury often develop long-term disabilities, resulting in significant losses of healthy life years, long after the acute injury. Most injury-related epidemiological studies have focused on using incidence, case fatality rates, or population mortality rates to describe the public health impact of injuries [3–5]. Considering that non-fatal consequences of injury vary widely in their severity and duration, and that premature mortality is an important injury consequence, it is of great importance to use a summary measure of population health that includes both mortality and morbidity when assessing the impact of injury.

A widely used population health indicator combining the impact of mortality and morbidity is the disability-adjusted life year (DALY) [6, 7]. The DALY – used in the Global Burden of Disease (GBD) study – quantifies the BoD by merging mortality, expressed in years of life lost (YLL) and morbidity, expressed in years lived with disability (YLD) into one single metric [7]. Historically, the BoD concept allows for both geographical and temporal comparisons of the impact of different diseases and injuries on population health [7, 8].

Many countries and public health agencies have adopted the DALY metric for monitoring population health and identifying priorities in preventive efforts; however, calculating the burden due to injuries is complex. It requires adequate epidemiological data from a range of administrative sources that include information on the *cause-of-injury*, which pertains to the intent and mechanism of injury, and the *nature-of-injury*, which pertains to the type of injury and the severity of their consequences [9]. Furthermore, calculating the burden due to injury requires many specific methodological choices, particularly for the non-fatal consequences [10, 11]. First, a choice has to be made as to whether incidence-based or prevalence-based injury YLDs are to be calculated [12]. Incidence-based YLD calculations capture the current and future BoD of incident cases and may be more useful to inform injury intervention strategies compared to prevalence-based calculations. Second, to assess injury YLDs, a methodological approach and data are required to inform short-term and long-term disability based on post-injury functional status. A third methodological choice relates to the set of disability weights (DWs) that is applied to injury-related health states. Several sets of DWs exist with ranging coverage of injury-related health states [13, 14].

Another methodological choice relates to the calculation of the YLLs. For the calculation of YLLs, information on the remaining life expectancy at age of death is needed and this is derived from aspirational or standard (i.e., observed global life expectancy) or national (i.e., national life expectancy) life tables. In BoD studies, the choice of the life table affects the magnitude of the YLL and as a result affects country and time-period comparability [15].

Driven by the disparity in the mortality and morbidity injury patterns across Europe, where many independent BoD studies have been published, there is a need to explore which injury BoD design choices have been applied over the years. Until now, an overview of the YLL and YLD design choices that have been used in BoD studies in injury populations is not available. Therefore, we aimed to identify existing injury BoD activities undertaken in Europe and to comprehensively review the methodological design choices and assumption parameters that have been used to calculate YLL and YLD in these studies. The following research questions were addressed:

- In which GBD European Region countries has injury BoD assessment been performed?
- Which YLD methodological design choices and assumption parameters have been made in single-country and multi-country injury BoD assessments?
- Which YLL methodological design choices and assumption parameters have been made in single-country and multi-country injury BoD assessments?

## Methods

The design of this systematic literature review follows the Preferred Reporting Items for Systematic Reviews and Meta-Analyses (PRISMA) 2020 statement [16]. The protocol can be found on PROSPERO under the registration number: CRD42020177477.

### Inclusion and exclusion criteria and injury definitions

In this literature review, we included studies that assessed the health outcomes from injury in terms of YLL, YLD, or DALY. Our review is limited to injury-specific BoD studies; we have excluded studies that reported on all-cause disease burden. All-cause BoD studies assess the impact of multiple causes covered by the three broad GBD cause hierarchy groups namely Group I “Communicable, maternal, neonatal, and nutritional diseases”, Group II “Non-communicable diseases”, and Group III “Injuries”. Injury-specific BoD studies assess the impact of the GBD cause-of-injury and/or nature-of-injury outcomes and did not assess YLL, YLD, or DALY resulting from Group I and/or Group II. Details of the GBD 2019 disease and injury hierarchical cause list can be found elsewhere [17]. We included only BoD studies conducted within the GBD European Region. A full list of these geographic locations can be found in the Additional file 1 (page 2). Since the DALY concept was introduced in the 1993 World Development Report [18], we screened only BoD studies published after January 1990.

We excluded disease burden studies that did not assess the impact of injury causes. We also excluded studies that quantified the magnitude of risk factor exposure, because methodological approaches for the risk factor assessment were beyond the scope of this review. Further, we excluded studies with outcomes other than YLL, YLD and/or DALY (e.g. computation of potential years of life lost, estimation of DWs), as well as citation-only books, theses, conference proceedings, editorials, and letters-to-editor.

We considered BoD studies that defined injury as a physical harm resulting from acute exposure to physical agents such as mechanical energy, electricity, heat, chemicals and radiation in amounts beyond the threshold of human tolerance [19]. We used the International Classification of Diseases (ICD) system to identify causes-of-injury, where the injury incidence and causes-of-death are defined in ICD-9 codes E000-E999 and ICD-10 chapters V–Y. Non-fatal consequences of injuries and poisonings are classified based on ICD-9 codes 800–999 and ICD-10 chapters S and T. Thus, we included studies assessing the injury burden in terms of nature-of-injury and cause-of-injury. We did not include psychological (e.g. post-traumatic stress disorder) or pathological consequences (e.g. osteoporotic fractures) resulting from a prior trauma. An overview of the GBD cause-nature categories can be found in the Additional file 1 (page 3).

### Data sources and search strategy

We searched for eligible BoD records on five main platforms: EMBASE, MEDLINE, Cochrane Central, Google Scholar, and Web of Science. An experienced librarian from the Erasmus MC Medical Library performed the search strategy on 2 April 2020, updating it on 6 May 2021. We did not set any language restrictions. Details of the systematic search strategy can be found in the Additional file 1 (page 5).

We examined the grey literature on: (a) OpenGrey, OAIster, CABDirect, and the World Health Organization (WHO) websites and (b) government and/or public health websites from the targeted European countries (see Additional file 1; page 8). We also asked the COST Action CA18218 members to identify further all-cause or injury-specific BoD sources. One researcher (PC) handsearched references of those eligible and included BoD records by looking into the references of published studies and reports.

### Screening and data extraction

We listed all the records obtained from the search strategy (phase 1) and the COST Action CA18218 participants (phase 2) on an EndNote X9 and Excel spreadsheet, respectively. After removing duplicates, we imported all the records on the EndNote X9 software. Two researchers (PC and VG) performed the screening. In essence, we selected eligible studies following three steps: title (first step) and abstract screening (second step), followed by our identifying potentially relevant studies and screening upon full-text (third step). Discussions with EP and the study supervisor (JH) resolved any doubts.

Two researchers (PC and EP) performed the extraction of data, independently of each other, using an Excel spreadsheet which included the following a priori information: first author, year of publication, country or region, study type, type of analysis, methodological choices regarding the YLL and YLD calculations, and injury-specific approaches for BoD calculations. The extracted items, followed by their definitions, can be found in the Additional file 1 (page 9). We piloted the data extraction grid for 5% of the included BoD studies with no masking, during this process. Data extraction for the non-English papers was performed by the *burden-eu* native speakers and discussed with PC. Finally, PC and EP compared, assessed, and discussed the data extraction forms. Discussions with the study supervisor (JH) resolved any disagreements.

### Study classifications

In this review, we classified studies according to the: (a) number of countries that were covered (single-country *versus* multi-country BoD study), (b) reported causes of ill-health (all-cause *versus* injury-specific BoD study) and (c) type of study (independent *versus* GBD-linked injury BoD study). The term *‘independent injury BoD study’* refers to single-country or multi-country studies for which researchers performed own calculations and analyses of YLL, YLD and/or DALY caused by injuries. The term *‘GBD-linked injury BoD study’* refers to single-country or multi-country studies that present GBD estimates or secondary analyses of GBD results. In this group, we also classified studies in which the injury YLL, YLD, and/or DALY estimates were derived from the WHO Global Health Estimates (GHE) [20], though the GHE and GBD are two separate repositories.

The following review focuses on the summary of single-country and multi-country independent and GBD-linked injury-specific BoD studies that have been performed across European countries over the 1990–2021 period. Descriptive analysis and the reference lists of the identified all-cause-related European BoD studies can be found in the Additional file 1 (page 12).

## Results

### Literature search

**Fig. 1:**
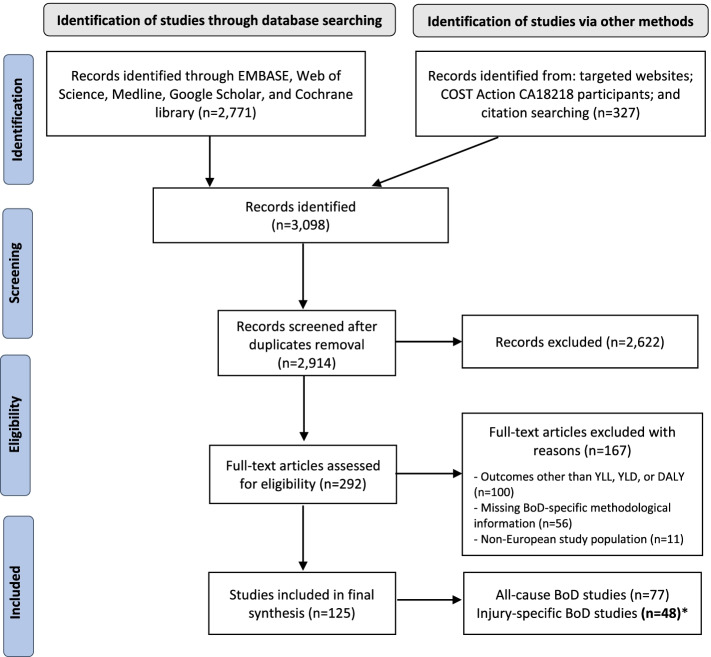
Flowchart of the literature search strategy of existing European burden of disease studies * This systematic literature review is limited to injury-specific BoD assessments undertaken across Europe; January 1990 - May 2021

We retrieved a total of 2,771 articles from the developed search strategy (EMBASE = 1,791; Web of Science = 560; MEDLINE via Ovid engine = 261; Google Scholar = 128; and Cochrane library via Wiley engine = 31). We identified 327 additional records via other methods (i.e., grey literature and citation handsearching). After removing duplicates, we screened a total of 2,914 records. We performed full-text screening for 292 BoD studies, and we extracted data from 125 BoD studies. Out of these 125 BoD studies, 48 performed an injury-specific disease burden assessment. Figure 1 shows the flowchart of the literature search strategy of existing disease burden studies and main reasons for exclusion.

### Study types per study classification and geographic location

**Fig. 2:**
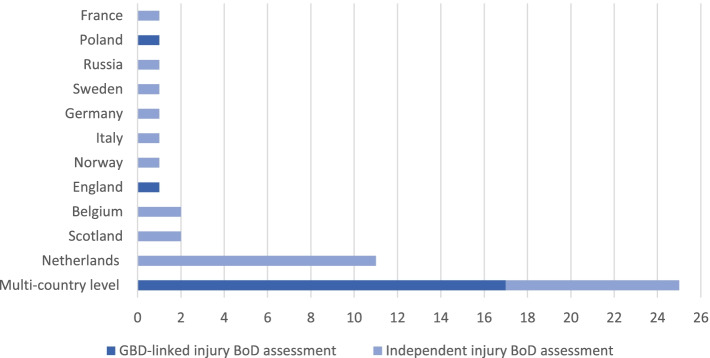
Number of GBD-linked and independent injury burden of disease studies per multi-country and single-country category

As described in Table 1 and Fig. 2, 40% (19 out of 48) consisted of GBD-linked studies, whereas 60% (29 out of 48) consisted of independent studies. Of the GBD-linked studies, 89% (17 out of 19) were multi-country studies and 11% (2 out of 19) were single-country studies. Of the independent studies, 28% (8 out of 29) were multi-country studies and 72% (21 out of 29) were single-country studies. Single-country injury disease burden assessments (*n* = 23) were performed in 11 European countries. The largest number of single-country independent studies was observed in the Netherlands (*n* = 11), followed by Scotland (*n* = 2), Belgium (*n* = 2), Germany (*n* = 1), Sweden (*n* = 1), Italy (*n* = 1), Norway (*n* = 1), France (*n* = 1), and Russia (*n* = 1). Two single-country studies undertaken in Poland (*n* = 1) and England (*n* = 1) assessed the burden of injuries using GBD results.

**Table 1:** Number of GBD-linked and independent single-country and multi-country studies

	***Injury-specific BoD studies (n* = *48)***	
	GBD-linked BoD assessments	Independent BoD assessments
Single-country	*n* = 2 (11%)	*n* = 21 (72%)
Multi-country	*n* = 17 (89%)	*n* = 8 (28%)

### Cause-of-injury *versus* nature-of-injury burden of disease studies

**Fig. 3:**
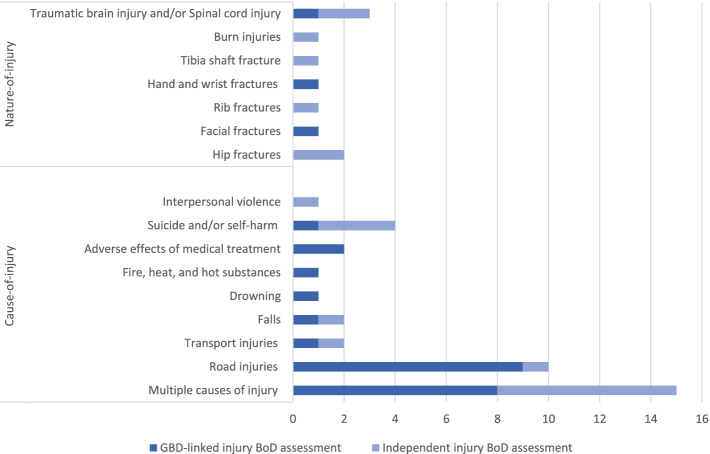
Number of GBD-linked and independent injury burden of disease studies (*n* = 48) by cause-nature of injury

Figure 3 illustrates the number of GBD-linked and independent injury BoD studies (*n* = 48) by cause-nature of injury. In total, 38 out of 48 studies reported the BoD by cause-of-injury category, and the remaining 10 studies reported the BoD by nature-of-injury category. The majority of the cause-of-injury BoD studies were GBD-linked studies (24 out of 38). Nine out of these 24 studies evaluated the impact of road injuries. In contrast, among the independent studies that reported cause-of-injury (14 out of 38), the number of multi-cause (7 out of 14) and suicide and/or self-harm (3 out of 14) studies stand out. Moreover, the number of independent studies that reported nature-of-injury (7 out of 10) was higher compared to the number of GBD-linked studies (3 out of 10). The largest number of independent nature-of-injury BoD studies assessed the impact of hip fractures (2 out of 7), and traumatic brain injury and/or spinal cord injury (2 out of 7).

### Classification of injury diagnosis

Single-country and multi-country GBD-linked studies (17 out of 19) re-ordered injury causes-of-death using the ICD-9 or ICD-10 coding system. Two of these studies (2 out of 19) did not report the injury classification scheme. Similarly, most single-country and multi-country independent BoD studies (82%) gathered injury diagnosis from the ICD code-system. Some of these studies (38%) translated injury diagnosis according to the EUROCOST classification system [21]. Three single-country and multi-country independent injury studies (11%) did not report the diagnosis classification system.

### YLD methodological choices in injury burden of disease studies

#### Prevalence-based versus Incidence-based calculations

Table 2 summarizes the methodological design choices and assumption parameters that have been used in injury BoD studies. Most single-country independent studies have followed the incidence-based approach to calculate YLDs due to injury [22–38]. Two independent injury BoD reports conducted in Scotland have performed own prevalence-based YLD calculations [39, 40]. Conversely, two single-country studies have evaluated the impact of injury using GBD results; a United Kingdom comparative report presented prevalence-based YLD calculations [41], and a Polish study quantified injury DALYs using a combination of Polish data on traffic fatalities and GBD 2010 data to assess the burden due to traffic injuries in Warsaw [42]. Seven multi-country independent studies quantified the burden of injury using the incidence-based approach [43–49]. Also, 11 multi-country GBD-linked studies estimated injury YLDs using the prevalence-based approach [1, 50–59]; of which 10 used GBD data as primary source of data and one of these studies used the 2015 WHO GHE as a primary source of data. Moreover, four out of the 11 multi-country GBD-linked studies followed an incidence-based approach to assess injury YLD. [60–63]. These four injury BoD studies were conducted before 2010.

**Table 2:** Methodological design choices and assumption parameters in injury burden of disease studies

Author	Year	Single- or multi-country category?	Geographic Location	Type of study		Injury classification		Classification of injury diagnosis	Design choices of YLL calculations	Design choices of YLD calculations	
				**Independent study**	**GBD-linked study**	**Cause-of-injury category**	**Nature-of-injury category**			**Incidence- or prevalence-based approach?**	**Usage of disability weights**
Aldridge et al. [50]	2017	Multi-country	WHO European Region		•	•		ICD-9; ICD-10	WHO standard model life tables	Prevalence	GBD DWs
Begg & Tomijima [60]	2006	Multi-country	Global			•	•	ICD-9; ICD-10	GBD standard model life tables	Incidence	GBD DWs
Crowe et al. [52]	2020	Multi-country	Global		•	•	•	ICD-9; ICD-10	NA	Prevalence	GBD DWs
Dhondt et al. [23]	2012	Single-country	Belgium (Flanders; Brussels)	•		•	•	ICD-9 (aggregated to the EUROCOST classification)	Belgian life table	Incidence	Empirical DWs; GBD DWs
Dhondt et al. [22]	2013	Single-country	Belgium (Flanders; Brussels)	•		•	•	ICD-9; ICD-10 (aggregated to the EUROCOST classification)	Belgian LE	Incidence	Dutch DWs; GBD DWs
Fattahov & Piankova [64]	2018	Single-country	Russia	•		•		ICD-10	GBD standard model life tables	NA	NA
Franklin et al. [65]	2020	Multi-country	Global		•			ICD-9; ICD-10	GBD standard model life tables	NA	NA
Gobbino et al. (on behalf of CRMSS) [24]	2012	Single-country	Italy (Friuli Venezia Giulia)	•		•	•	ICD-9	Italian life table	Incidence	Australian DWs
Haagsma et al. [25]	2008	Single-country	Netherlands	•		•		ICD-9 (aggregated to the EUROCOST classification)	NA	Incidence	Empirical DWs
Haagsma et al. [43]	2012	Multi-country	Netherlands; Ceres; Thailand	•		•		ICD-10 (aggregated to the EUROCOST classification)	Standard West 26	Incidence	Empirical DWs
Haagsma et al. [1]	2016	Multi-country	Global		•	•	•	ICD-9; ICD-10	GBD standard model life tables	Prevalence	GBD DWs
Haagsma et al. [54]	2020	Multi-country	GBD Western Europe		•	•		ICD-9; ICD-10	GBD standard model life tables	Prevalence	GBD DWs
Haagsma et al. [53]	2020	Multi-country	Global		•	•		ICD-9; ICD-10	GBD standard model life tables	Prevalence	GBD DWs
Hagen et al. [26]	2020	Single-country	Norway	•			•	NR	GBD standard model life tables	Incidence	GBD DWs
Hoeymans & Schoemaker [38]	2010	Single-country	Netherlands	•		•	•	ICD-10	Dutch life table	Incidence	Empirical DWs
Holtslag et al. [27]	2008	Single-country	Netherlands	•		•		NR	Dutch life table	Incidence	Empirical DWs
James et al. [55]	2019	Multi-country	Global		•	•	•	ICD-9; ICD-10	GBD standard model life tables	Prevalence	GBD DWs
James et al. [11]	2019	Multi-country	Global		•	•	•	ICD-9; ICD-10	NA	Prevalence	GBD DWs
James et al. [56]	2020	Multi-country	Global		•	•	•	ICD-9; ICD-10	NA	Prevalence	GBD DWs
Johnell & Kanis [44]	2004	Multi-country	World Bank Regions	•			•	NR	NR	Incidence	GBD DWs
Khan et al. [57]	2020	Multi-country	Global		•	•		ICD-9; ICD-10	GBD standard model life tables	Prevalence	GBD DWs
Lalloo et al. [58]	2020	Multi-country	Global		•	•	•	ICD-9; ICD-10	GBD standard model life tables	Prevalence	GBD DWs
Lapostolle et al. [28]	2009	Single-country	France	•		•	•	ICD-10 (AIS codes)	French LE	Incidence	GBD DWs
Leliveld et al. [29]	2020	Single-country	Netherlands	•			•	ICD-9; ICD-10	NA	Incidence	Empirical DWs
Lin [59]	2016	Multi-country	Global		•	•		NR	GBD standard model life tables	Prevalence	GBD DWs
Lukaschek et al. [66]	2012	Single-country	Germany	•		•		ICD-10	German LE	NA	NA
Lunevicius & Haagsma [41]	2018	Single-country	England (9 English Regions)		•	•		ICD-9; ICD-10	GBD standard model life tables	Prevalence	GBD DWs
Lyons et al. [45]	2017	Multi-country	EU-28	•		•	•	ICD-10	GBD standard model life tables	Incidence	Empirical DWs
Majdan et al. [67]	2017	Multi-country	EU-16	•		•	•	ICD-10	European Union life table	NA	NA
Naghavi et al. [68]	2019	Multi-country	Global		•	•		ICD-9; ICD-10	GBD standard model life tables	NA	NA
NHS Health Scotland [39]	2016	Single-country	Scotland	•		•	•	ICD-10	Scottish life table	Prevalence	GBD DWs
NHS Health Scotland [40]	2016	Single-country	Scotland	•		•	•	ICD-10	Scottish life table	Prevalence	GBD DWs
Peden et al. [61]	2002	Multi-country	Global		•	•	•	ICD-9; ICD-10	GBD standard model life tables	Incidence	GBD DWs
Polinder et al. [47]	2007	Multi-country	Austria; Denmark; UK (England & Wales); Ireland; Norway; Netherlands	•		•	•	ICD-9; ICD-10	GBD standard model life tables	Incidence	GBD DWs
Polinder et al. [46]	2010	Multi-country	Austria; Latvia; Denmark; UK (England & Wales); Ireland; Netherlands; Norway; Slovenia	•		•	•	ICD-9; ICD-10	GBD standard model life tables	Incidence	GBD DWs; Empirical DWs
Polinder et al. [31]	2012	Single-country	Netherlands	•		•		ICD-9 (aggregated to the EUROCOST classification)	GBD standard model life tables	Incidence	Empirical DWs
Polinder et al. [30]	2015	Single-country	Netherlands	•		•		ICD-9 (MAIS code; aggregated to the EUROCOST classification)	GBD standard model life tables	Incidence	Empirical DWs
Prins et al. [32]	2021	Single-country	Netherlands	•			•	NA	NA	Incidence	Empirical DWs
Scholten et al. [33]	2014	Single-country	Netherlands	•			•	ICD-9	GBD standard model life tables	Incidence	Empirical DWs
Snijders et al. [34]	2016	Single-country	Netherlands	•		•	•	ICD-9	Dutch life table	Incidence	Empirical DWs
Sethi et al. [62]	2008	Multi-country	WHO European Region		•	•		ICD-9; ICD-10	GBD standard model life tables	Incidence	GBD DWs
Spronk et al. [48]	2020	Multi-country	Netherlands; New Zealand; Australia	•			•	NA	NA	Incidence	Empirical DWs
Tainio et al. [35]	2014	Single-country	Sweden	•		•	•	ICD-9; ICD-10 (AIS code)	GBD standard model life tables	Incidence	GBD DWs
Tainio [42]	2015	Single-country	Poland		•	•		NR	NR	Polish data on traffic fatalities and GBD 2010 data	NR
Twisk et al. [36]	2017	Single-country	Netherlands	•		•		ICD-9 (aggregated to the EUROCOST classification)	Dutch LE	Incidence	Empirical DWs
Valent et al. [63]	2004	Multi-country	WHO European Region		•	•		ICD-9; ICD-10	GBD standard model life tables	Incidence	GBD DWs
Weijermars et al. [37]	2016	Single-country	Netherlands	•		•		ICD-9 (aggregated to the EUROCOST classification)	NA	Incidence	Empirical DWs
Weijermarset al. [49]	2018	Multi-country	Austria; Spain; Belgium; France; England; Netherlands	•		•	•	ICD-9; ICD-10; aggregated to the EUROCOST classification)	NA	Incidence	Empirical DWs

*AIS* Abbreviated Injury Scale, *BoD* Burden of Disease, *CRMSS* Centro regionale di monitoraggio della sicurezza stradale, *DALY* Disability-Adjusted Life Years, *DW* Disability Weight, *EUROCOST* EUROCOST classification of injuries, *GBD* Global Burden of Disease, *ICD* International Classification of Diseases, *LE* Life Expectancy, *MAIS* Maximum Abbreviated Injury Scale, *NA* Not Applicable, *NR* Not Reported, *UK* United Kingdom, *YLD* Years-Lived with Disability, *YLL* Years of Life Lost due to premature mortality, *WHO* World Health Organization

#### Use of disability weights

Several sets of DWs were used to assess injury BoD estimates in independent studies. More than half (56%) of these studies, applied empirical DWs [25, 27, 29–34, 36–38, 43, 45, 48, 49]. All independent studies that used empirical DWs have performed incidence-based YLD calculations. Seven single-country independent injury BoD studies used GBD DWs [26, 28, 35, 39, 40, 44, 47], three used a combination of DWs [22, 23, 46], and one study applied Australian DWs [24].

### YLL methodological choices in injury burden of disease studies

#### Choice of life table

Most single-country independent studies have used national life tables [23, 24, 27, 33, 38–40] or national life expectancies [22, 28, 36, 66] to calculate YLLs. The remaining single-country independent BoD studies used aspirational model life tables that have a standard life expectancy at birth, such as those used in the GBD study [26, 30, 31, 33, 35, 64]. Multi-country independent studies frequently used aspirational global [43, 45–47] or European [67] life tables. The remaining single-country and multi-country GBD-linked BoD studies used the standard model life tables from GBD/WHO [1, 41, 50, 51, 53–63, 65, 68].

## Discussion

This systematic literature review has provided insights into the methodological design choices and assumption parameters that have been used to quantify the burden of injury in terms of YLL, YLD, or DALY. A total of 48 BoD studies met our inclusion criteria; more than half being single-country or multi-country independent studies, while the remaining were GBD-linked studies. Considerable methodological variation across injury BoD studies was observed; differences were mainly apparent in the design choices or assumption parameters towards injury YLD calculations, implementation of DWs, and the choice of life table for YLL calculations.

First, considerable heterogeneity exists in the aggregation level of cause-of-injury and nature-of-injury categories (see Fig. 3) that were used in the calculations and reporting of burden of injury studies. Among the unintentional injury-specific assessments, we observed a high number of falls-related BoD studies and no injury disease burden assessments at all related to exposure to mechanical forces, poisonings, or foreign body and animal contact. Moreover, there was diversity in the cause-of-injury and nature-of-injury categories reported. Most studies calculated DALYs for multiple causes-of-injury, yet there were also several studies that were limited to one specific nature-of-injury category, such as traumatic brain injury, or cause-of-injury category, such as road injury.

The high percentage of studies quantifying the burden of road injury has enhanced the visibility of road injury in Europe and shown that (injury) BoD assessments can, in turn, inform health policy and measures. Burden of road injury studies can be used to monitor the possible effect of improvements in car safety technologies, road infrastructure, better compliance with speed limits or seat-belt or helmet use, as observed across most European countries [69, 70]. For instance, there significant decline in road injury mortality and DALY rates across the European sub-regions over the 2000–2019 period [71].

Another striking finding of our systematic review was that studies that reported on nature-of-injury DALYs were more often independent studies than GBD-linked ones. A possible explanation for this finding may be that nature-of-injury DALYs were available from the GBD 2013 study onwards [72]. Before that, only cause-of-injury DALYs were available from the GBD results tool. The burden of injury studies that were limited to one specific cause-of-injury were focused on those causes-of-injury that are listed in the top 10 ranking of injury DALYs in Europe [55].

Second, our review reveals that most independent injury BoD studies (78%) were performed in Western European countries, while the number of injury disease burden studies across Central and Eastern European countries was limited. A possible explanation for this difference may be the lack of appropriate data sources, harmonization of data collection processes, a decentralized system of records access and poor-quality checks in the Central and Eastern European region compared to the Western European region. A second explanation may be that the use of these health metrics as indicators of health status may not be valued as important in these countries and their health reporting systems. This issue, in combination with the lack of resources, capacity or expertise in the use of these BoD metrics, contribute further to the chasm between data availability, data quality checking and subsequent data use for such large-scale national disease data estimation studies. Also, a variety of injury preventive interventions and/or policies has been developed in many Western European countries [73, 74]. Hence, many of the injury premature deaths and disabilities occur in Central and Eastern Europe [17], where fewer countries had developed national policies for injury prevention [74–76]. Future injury BoD assessments may be important in facilitating decision-making processes for injury policy formulation in these European regions.

Third, while most of injury BoD studies used the ICD coding system to classify injuries, we found that some independent BoD studies classified injury consequences based on the 39 injury-diagnoses of the EUROCOST system [21]. This classification system was developed for assessments of the cost of illness of injury [21, 77, 78] and may be less appropriate for injury DALY calculations due to nature-of-injury groupings encompassing injuries that vary widely in severity and duration. Significantly, some single-country independent studies did not report the injury diagnosis coding system or the methods that were used to deal with inaccurately coded injury deaths. This highlights the need for development and use of guidelines for performing and reporting of injury BoD studies.

Fourth, we found that most independent BoD studies used the incidence-based approach to estimate injury YLDs. This is at odds with the GBD approach (i.e., prevalence-based), which applies a meta-regression tool (DisMod-MR) to stream out (long-term) prevalence for each combination of cause-of-injury and nature-of-injury from incidence, by assuming a steady state where rates are consistently stable over time [11, 17]. The choice of incidence *versus* prevalence approach should be dictated by the pre-defined goals and application of the study. For instance, when assessing the burden of injury in terms of DALY and its components and planning, implementing or evaluating preventive strategies, an incidence-based approach should be used, whereas for health services planning purposes, a prevalence-based approach might be more appropriate.

Fifth, most single-country independent injury BoD studies used national life tables to calculate YLLs. The choice between national and global aspirational life-table is dependent on the study scope [15]. Aspirational life-tables ensure internationally comparable results since they are based on the same population structure, while national life-tables preclude the possibility of cross-country comparisons.

Furthermore, we observed that some injury BoD studies did not report the life-table that had been used to calculate YLLs. This suggests a need for improvements in the reporting of future injury disease burden studies, as the choice of national or aspirational life-table is crucial when performing a BoD assessment; evidence has illustrated the impact of how ranking of causes is affected [79]. The use of standardized reporting guidelines in DALY calculation studies would enhance comparability of results, communication among BoD researchers and/or policy makers, as well as facilitate quality assessments of the disease burden studies.

Lastly, a crucial methodological step in causes-of-death analysis is the estimation of total all-cause mortality (also referred to as mortality envelope) by each age and sex strata, for correcting death under-counting or over-counting using either redistribution methods and/or regression techniques etc. Although insight into this methodological step was beyond the scope of our systematic literature review, future studies should investigate whether mortality envelopes are used in disease burden studies, and if they are used, which methods are applied to construct them.

### Strengths and limitations of the study

This systematic literature review may be limited by the nature of the grey literature searched and the national public health websites targeted. Although we have used a variety of literature databases and search engines, some BoD studies may have been missed. However, it is possible that other BoD studies estimating the burden of injuries have been conducted but not published or documented. Despite these limitations, our systematic literature review provides the first of its kind in bringing together existing injury-specific BoD studies undertaken in Europe. We comprehensively reviewed the methodological design choices and assumption parameters that have been made to calculate YLL, YLD, and DALY in these European studies since the 1990s. Finally, we sought to provide recommendations with regard to the application and reporting of (injury) YLL and YLD design choices.

## Conclusions

In this systematic literature review we examined independent and GBD-linked studies that assessed the burden caused by injury, in European Region countries. Considerable methodological variation across injury BoD assessments was observed; differences were mainly apparent in the design choices or assumption parameters towards injury YLD calculations, implementation of DWs, and the choice of life table for YLL calculations. Development and use of guidelines for performing and reporting of BoD studies is crucial to enhance transparency and comparability of injury BoD estimates across Europe and beyond.

## Supplementary Information

**Additional file 1.** Search strategy and grey literature search and overview of studies.

**Additional file 2.** Data extraction form. 

## Data Availability

All data generated or analyzed during this study are publicly available at the cited links, and also at the Appendices.

## Abbreviations

BoD: Burden of Disease
DALY: Disability-adjusted life years
DW: Disability Weight
GBD: Global Burden of Disease
ICD: International Classification of Diseases
YLD: Years lived with disability
YLL: Years of life lost
