# Supplementary material for: Methodological considerations in injury burden of disease studies across Europe: a systematic literature review
Source: BMC Public Health. 2022 Aug 17;22:1564. doi: 10.1186/s12889-022-13925-z (PMC9382747; doi:10.1186/s12889-022-13925-z)
Supplement: Supplementary file 1 — Additional file 1. Search strategy and grey literature search and overview of studies. [file 12889_2022_13925_MOESM1_ESM.docx]

**Additional file**

**Methodological considerations in injury burden of disease studies across Europe: a systematic literature review**

**Chapter 1**

- 1. Global Burden of Disease ‘European Region countries’ ………………………………………………….………….. 2
  2. Overview of GBD injury causes …………………………….……………….….…………….…………………………………. 3
  3. Search strategy ………………………………………………………………………………………………………………………….. 5
  4. Grey literature search and websites of targeted national public health agencies ……….……………… 8
  5. Definitions of the used data extraction items ……………………………………….………………………………….… 9

**Chapter 2**

Systematic review analysis of all-cause BoD studies …….…………………………………….…………………………… 12

**Chapter 3**

Reference list of the included injury-specific and all-cause disease burden studies …………………………. 15

**Chapter 1**

- 1. **Global Burden of Disease ‘European Region countries’**

We systematically searched burden of disease (BoD) assessments undertaken across the Global Burden of Disease (GBD) European region countries. Full list of the GBD European region countries can be found below:

1. GBD Central Europe: *Albania, Bosnia and Herzegovina, Bulgaria, Croatia, Czechia, Hungary, Montenegro, North Macedonia, Poland, Romania, Serbia, Slovakia, and Slovenia*
2. GBD Eastern Europe: *Belarus, Estonia, Latvia, Lithuania, Republic of Moldova, Russian Federation, and Ukraine*
3. GBD Western Europe: *Andorra, Austria, Belgium, Cyprus, Denmark, Finland, France, Germany, Greece, Iceland, Ireland, Israel, Italy, Luxembourg, Malta, Monaco, Netherlands, Norway, Portugal, San Marino, Spain, Sweden, Switzerland, and the United Kingdom*

The World Health Organization (WHO) Regional Office for Europe classifies Turkey within Europe. Please note that, in our analysis we decided to include Turkish BoD studies as part of Western Europe. Moreover, disease burden studies performed at the constituent country or territory level were also considered. For instance, BoD studies that have been conducted in Greenland are categorized as Danish BoD assessments.

- 1. **Overview of GBD causes-nature of injury**

Selection of causes-of-injury and nature-of-injury was made according to the GBD 2019 study. Case definitions and ICD-codes of each of the cause-of-injury and nature-of-injury categories can be found elsewhere [1].

| **Global Burden of Disease causes-of-injury** | **Global Burden of Disease nature-of-injury** |
| --- | --- |
| **Transport injuries** | Amputation of lower limbs, bilateral |
| Road injuries | Amputation of upper limbs, bilateral |
| Pedestrian road injuries | Amputation of fingers (excluding thumb) |
| Cyclist road injuries | Amputation of lower limb, unilateral |
| Motorcyclist road injuries | Amputation of upper limb, unilateral |
| Motor vehicle road injuries | Amputation of thumb |
| Other road injuries | Amputation of toe/toes |
| Other transport injuries | Lower airway burns |
| **Unintentional injuries** | Burns, <20% total burned surface area without lower airway burns |
| Falls  Drowning  Fire, heat and hot substances | Burns, ≥20% total burned surface area or ≥10% burned surface area if head/neck or hands/wrist involved without lower airway burns |
| Poisonings | Fracture of clavicle, scapula or humerus |
| Poisoning by carbon monoxide | Fracture of face bones |
| Poisoning by other means | Fracture of foot bones except ankle |
| Exposure to mechanical forces | Fracture of hand (wrist and other distal part of hand) |
| Unintentional firearm injuries | Fracture of hip |
| Unintentional suffocation | Fracture of patella, tibia or fibula or ankle |
| Other exposure to mechanical forces | Fracture of pelvis |
| Adverse effects of medical treatment | Fracture of radius and/or ulna |
| Animal contact | Fracture of skull |
| Venomous animal contact | Fracture of sternum and/or fracture of one or more ribs |
| Non-venomous animal contact | Fracture of vertebral column |
| Foreign body | Fracture of femur, other than femoral neck |
| Pulmonary aspiration and foreign body in airway | Minor Traumatic Brain Injury |
| Foreign body in eyes | Moderate/severe Traumatic Brain Injury |
| Foreign body in other body part  Environmental heat and cold exposure | Spinal cord lesion at neck level  Spinal cord lesion below neck level |
| Other unintentional injuries | Muscle and tendon injuries, including sprains and strains lesser dislocations |
|  | Foreign body in ear |
|  | Open wound(s) |
| *(continued from the page)* | |
| **Global Burden of Disease causes-of-injury** | **Global Burden of Disease nature-of-injury** |
| **Self-harm and interpersonal violence** | Contusion in any part of the body |
| Self-harm | Superficial injury of any part of the body |
| Self-harm by firearm | Dislocation of hip |
| Self-harm by other specified means | Dislocation of knee |
| Interpersonal violence | Dislocation of shoulder |
| Assault by firearm | Foreign body in respiratory system |
| Assault by sharp object | Foreign body in gastrointestinal and urogenital system |
| Assault by other means | Drowning and non-fatal submersion |
| **Forces of nature, conflict and terrorism, and executions and police conflict** | Asphyxiation  Crush injury |
| Exposure to forces of nature | Nerve injury |
| Conflict and terrorism | Injury to eyes |
| Executions and police conflict | Poisoning requiring urgent care |
|  | Severe chest injury |
|  | Internal haemorrhage in abdomen and pelvis |
|  | Effect of different environmental factors |
|  | Complications following therapeutic procedures |
|  | Multiple fractures, dislocations, crashes, wounds, pains and strains |
| James, S.L., et al., Estimating global injuries morbidity and mortality: methods and data used in the Global Burden of Disease 2017 study. *Inj Prev*, 2020. 26(Supp 1): p. i125-i153 | |

- 1. **Search Strategy**

**Embase**

('disability-adjusted life year'/de OR (DALY OR DALYs OR ((disabil*) NEAR/4 (adjust*) NEAR/4 (life*) NEAR/4 (year*)) OR YLL OR YLLs OR ((year*) NEXT/2 (life*) NEXT/1 (lost*)) OR YLD OR YLDs OR ((year*) NEAR/3 (lived) NEAR/3 (disabil*))):ab,ti,kw) AND ('Europe'/exp OR 'Yugoslavia'/de OR 'Israel'/de OR 'European Union'/de OR 'European'/de OR 'EU citizen'/de OR (europ* OR austria* OR belgium OR belgian* OR Denmark OR danish OR france OR french* OR german* OR ireland OR irish* OR italy OR italian* OR luxemb* OR netherlands OR dutch OR norway OR sweden OR switzerland OR swiss OR united-kingdom OR albania OR armenia OR bosnia* OR herzegovin* OR bulgar* OR croatia* OR cyprus OR czechoslovakia* OR estonia* OR finland OR georgia OR greece OR hungar* OR iceland* OR israel* OR kosov* OR latvia* OR lithuan* OR macedoni* OR malta OR montenegr* OR poland OR polish OR portug* OR romani* OR rumani* OR serbi* OR slovak* OR sloven* OR spain* OR spanish OR turkey* OR mediterran* OR czech* OR england* OR UK OR scotland OR wales OR britain* OR holland* OR scandinav* OR nordic-countr* OR yugoslov* OR baltic* OR flander* OR wallon* OR benelux* OR greek* OR andorra* OR azerbaijan* OR belarus* OR byelarus* OR byelorus* OR white-russia* OR monaco* OR moldova* OR moldovia* OR russian-federat* OR san-marin* OR ukrain*):ab,ti,kw)

**Medline**

((DALY OR DALYs OR ((disabil*) ADJ4 (adjust*) ADJ4 (life*) ADJ4 (year*)) OR YLL OR YLLs OR ((year*) ADJ2 (life*) ADJ (lost*)) OR YLD OR YLDs OR ((year*) ADJ3 (lived) ADJ3 (disabil*))).ab,ti,kf.) AND (exp Europe/ OR Yugoslavia/ OR Israel/ OR European Union/ OR (europ* OR austria* OR belgium OR belgian* OR Denmark OR danish OR france OR french* OR german* OR ireland OR irish* OR italy OR italian* OR luxemb* OR netherlands OR dutch OR norway OR sweden OR switzerland OR swiss OR united-kingdom OR albania OR armenia OR bosnia* OR herzegovin* OR bulgar* OR croatia* OR cyprus OR czechoslovakia* OR estonia* OR finland OR georgia OR greece OR hungar* OR iceland* OR israel* OR kosov* OR latvia* OR lithuan* OR macedoni* OR malta OR montenegr* OR poland OR polish OR portug* OR romani* OR rumani* OR serbi* OR slovak* OR sloven* OR spain* OR spanish OR turkey* OR mediterran* OR czech* OR england* OR UK OR scotland OR wales OR britain* OR holland* OR scandinav* OR nordic-countr* OR yugoslov* OR baltic* OR flander* OR wallon* OR benelux* OR greek* OR andorra* OR azerbaijan* OR belarus* OR byelarus* OR byelorus* OR white-russia* OR monaco* OR moldova* OR moldovia* OR russian-federat* OR san-marin* OR ukrain*).ab,ti,kf.)

**Cochrane**

((DALY OR DALYs OR ((disabil*) NEAR/4 (adjust*) NEAR/4 (life*) NEAR/4 (year*)) OR YLL OR YLLs OR ((year*) NEXT/2 (life*) NEXT/1 (lost*)) OR YLD OR YLDs OR ((year*) NEAR/3 (lived) NEAR/3 (disabil*))):ab,ti) AND ((europ* OR austria* OR belgium OR belgian* OR Denmark OR danish OR france OR french* OR german* OR ireland OR irish* OR italy OR italian* OR luxemb* OR netherlands OR dutch OR norway OR sweden OR switzerland OR swiss OR united-kingdom OR albania OR armenia OR bosnia* OR herzegovin* OR bulgar* OR croatia* OR cyprus OR czechoslovakia* OR estonia* OR finland OR georgia OR greece OR hungar* OR iceland* OR israel* OR kosov* OR latvia* OR lithuan* OR macedoni* OR malta OR montenegr* OR poland OR polish OR portug* OR romani* OR rumani* OR serbi* OR slovak* OR sloven* OR spain* OR spanish OR turkey* OR mediterran* OR czech* OR england* OR UK OR scotland OR wales OR britain* OR holland* OR scandinav* OR nordic-countr* OR yugoslov* OR baltic* OR flander* OR wallon* OR benelux* OR greek* OR andorra* OR azerbaijan* OR belarus* OR byelarus* OR byelorus* OR white-russia* OR monaco* OR moldova* OR moldovia* OR russian-federat* OR san-marin* OR ukrain*):ab,ti)

**Web of Science**

TS=(((DALY OR DALYs OR ((disabil*) NEAR/4 (adjust*) NEAR/4 (life*) NEAR/4 (year*)) OR YLL OR YLLs OR ((year*) NEAR/2 (life*) NEAR/1 (lost*)) OR YLD OR YLDs OR ((year*) NEAR/2 (lived) NEAR/2 (disabil*)))) AND ((europ* OR austria* OR belgium OR belgian* OR Denmark OR danish OR france OR french* OR german* OR ireland OR irish* OR italy OR italian* OR luxemb* OR netherlands OR dutch OR norway OR sweden OR switzerland OR swiss OR united-kingdom OR albania OR armenia OR bosnia* OR herzegovin* OR bulgar* OR croatia* OR cyprus OR czechoslovakia* OR estonia* OR finland OR georgia OR greece OR hungar* OR iceland* OR israel* OR kosov* OR latvia* OR lithuan* OR macedoni* OR malta OR montenegr* OR poland OR polish OR portug* OR romani* OR rumani* OR serbi* OR slovak* OR sloven* OR spain* OR spanish OR turkey* OR mediterran* OR czech* OR england* OR UK OR scotland OR wales OR britain* OR holland* OR scandinav* OR nordic-countr* OR yugoslov* OR baltic* OR flander* OR wallon* OR benelux* OR greek* OR andorra* OR azerbaijan* OR belarus* OR byelarus* OR byelorus* OR white-russia* OR monaco* OR moldova* OR moldovia* OR russian-federat* OR san-marin* OR ukrain*)))

**Google Scholar**

"disability adjusted life years" europe|france|germany|italy|netherlands|norway|sweden|switzerland|"united kingdom"|finland|greece|hungaria|israel|poland|portugal|romania|spain|turkey|england|britain|scandinavia

- 1. **Grey literature search and websites of targeted national public health agencies**

*Grey literature search engines*

| - OpenGrey: [www.opengrey.eu](http://www.opengrey.eu) | - **CABDirect:** [www.cabdirect.org](http://www.cabdirect.org) |
| --- | --- |
| - OAIster: <http://oaister.worldcat.org> | - **World Health Organization:** [www.who.int](http://www.who.int) |

*Websites of targeted national public health agencies*

| - Albania: [www.ishp.gov.al](http://www.ishp.gov.al) | - **Malta:** [www.deputyprimeminister.gov.mt/](http://www.deputyprimeminister.gov.mt/) |
| --- | --- |
| - Andorra: [www.salut.ad](http://www.salut.ad) | - **Montenegro:** [www.ijzcg.me/](http://www.ijzcg.me/) |
| - Austria: [goeg.at/](https://goeg.at/) | - **Netherlands:** [www.rivm.nl](http://www.rivm.nl/) |
| - Belarus: [minzdrav.gov.by/en/](http://minzdrav.gov.by/en/) | - **North Macedonia:** [www.iph.mk](http://www.iph.mk/) |
| - Belgium: [www.sciensano.be/en](http://www.sciensano.be/en) | - **Norway:** [www.fhi.no](http://www.fhi.no/) |
| - Bulgaria: [ncpha.government.bg](http://ncpha.government.bg) | - **Poland:** [www.pzh.gov.pl](http://www.pzh.gov.pl/) |
| - Cyprus: [www.moh.gov.cy/](http://www.moh.gov.cy/) | - **Portugal:** [www.dgs.pt](http://www.dgs.pt); [www.sns.gov.pt](http://www.sns.gov.pt) |
| - Czech Republic: [www.szu.cz](http://www.szu.cz) | - **Republic of Moldova:** [www.msmps.gov.md/](http://www.msmps.gov.md/) |
| - Denmark: [www.si-folkesundhed.dk](http://www.si-folkesundhed.dk) | - **Romania:** [www.insp.gov.ro/](https://www.insp.gov.ro/) |
| - Estonia: [www.tai.ee](http://www.tai.ee) | - **Russian Federation:** [www.minzdrav.gov.ru](http://www.minzdrav.gov.ru) |
| - Finland: [www.thl.fi/en/](http://www.thl.fi/en/) | - **Serbia:** [www.batut.org.rs/english.html](http://www.batut.org.rs/english.html) |
| - France: [www.santepubliquefrance.fr](http://www.santepubliquefrance.fr) | - **Slovakia:** [www.uvzsr.sk/en/](http://www.uvzsr.sk/en/) |
| - Germany: [www.rki.de](http://www.rki.de/EN) | - **Slovenia:** [www.nijz.si](http://www.nijz.si/) |
| - Greece: [www.statistics.gr](http://www.statistics.gr); [www.eody.gov.gr](http://www.eody.gov.gr) | - **Spain:** [www.isciii.es](http://www.isciii.es) |
| - Hungary: [www.nnk.gov.hu/](http://www.nnk.gov.hu/) | - **Sweden:** [www.folkhalsomyndigheten.se](http://www.folkhalsomyndigheten.se/) |
| - Iceland: [www.landlaeknir.is](http://www.landlaeknir.is/) | - **Switzerland:** [www.bag.admin.ch/bag/de/home.html](http://www.bag.admin.ch/bag/de/home.html) |
| - Ireland: [www.publichealth.ie](http://www.publichealth.ie/) | - **Turkey:** [www.saglik.gov.tr/?_Dil=2](http://www.saglik.gov.tr/?_Dil=2) |
| - Israel: [www.gov.il/](http://www.gov.il/) | - **Ukraine:** [en.moz.gov.ua/](https://en.moz.gov.ua/) |
| - Italy: [www.iss.it/](http://www.iss.it/)  - **Latvia:** [www.rsu.lv/en/institute-public-health](https://www.rsu.lv/en/institute-public-health)  - **Lithuania:** [sam.lrv.lt/en/](https://sam.lrv.lt/en/)  - **Luxembourg:** [www.lih.lu/](https://www.lih.lu/) | - **United Kingdom (Scotland):** [www.gov.uk/government/organisations/public-health-england](https://www.gov.uk/government/organisations/public-health-england); [www.scotpho.org.uk/](http://www.scotpho.org.uk/) |

- 1. **Definitions of the data extraction items used**

| General Information | PMID | A unique identifier number which assigned to a specific reference/article in the PubMed website. |
| --- | --- | --- |
|  | Journal | The name of the journal that published the selected article. |
|  | Title | The full title of the selected article. |
|  | Author(s) | List of author(s) with the use of **Vancouver style**. |
|  | Year | The year that the selected burden of disease (BoD) study was published. |
|  | Funding body  *(optional)* | A type of funding source, i.e.: government, institutional administrators, private industry, foundations, professional organisation, etc. |
|  | Language | The written language of the BoD study |
| Study characteristics | Cause of ill health | A single disease related to communicable or non-communicable disease or injury or an aggregation of diseases and injuries.  i.e., Non-communicable diseases (NCDs) [Yes/No]; Communicable diseases (CDs) [Yes/No]; Injuries [Yes/No]  Define as **injury-specific** or **all-cause BoD assessment** |
|  | Type of study | **Independent study** (i.e., single-country or multi-country studies for which researchers performed own calculations and analyses of years of life lost (YLL), years –lived with disability (YLD) and/or disability-adjusted life years (DALY)) *versus* **GBD-linked study** (i.e., single-country or multi-country studies that present Global Burden of Disease (GBD) estimates or secondary analyses of GBD results. |
|  | Reference population | Population whose health causes during some period of time is the source of the study data. Define as **single-country** or **multi-county BoD assessment** |
|  | Reference year | The year for which an estimate of incidence/prevalence/BoD is reported. |
|  | Stratification | The specific cause(s), and/or disease(s), and/or risk factor(s) related to mortality or disability indicators stratified for each year, age, and sex. [Yes/No] |
| Data input sources  Data input sources  *(continued)* | Data source mortality/YLL | *Were data sources that were used to derive mortality/YLL data specified by the authors?* [Yes/No]  *Relevant approaches for mortality data:* National statistics, disease registries, registry of death, survey data, vital registration systems, verbal autopsies, death registration systems, published literature, etc. |
|  | Mortality/YLL:  data integration | *Were multiple data sources integrated to arrive at the mortality/YLL data?* [Yes/No] |
|  | Data source incidence/prevalence/YLD  Data source incidence/prevalence/YLD  *(continued)* | *Were data sources that were used to derive incidence/prevalence/YLD data specified by the authors?* [Yes/No]  *Relevant data sources for morbidity data:* Police records, health service encounter data, emergency department visits, hospital admissions, etc.  *Other relevant data sources for morbidity data:* Published literature, disease registries, routine administrative and survey datasets, surveillance systems, health facility data, etc |
|  | Incidence/prevalence/YLD: data integration | *Were multiple data sources integrated to arrive at the incidence/prevalence/YLD data?* [Yes/No] |
| Data adjustments | Mortality/YLL:  data adjustment | [Yes/No] |
|  | Incidence/prevalence/YLD: data adjustment | [Yes/No] |
|  | Internal consistency | *Were adjustments made to ensure that the sum of cause‐specific mortality or impairments equals all‐cause mortality or impairments?* |
|  | Use of DisMod | DisMod is s a software tool that may be used to check the consistency of estimates of incidence, prevalence, duration and case fatality for diseases.  *Did the authors mention the use of DisMoD?* [Yes/No] |
| DALY method  DALY method  *(continued)* | Perspective of YLD estimates | - *Prevalence-based* perspective takes point prevalence measures of disability, adjusted for seasonal variation. - *Incidence-based* perspective captures the BoD in new diagnostic cases during a reference time-period and links all possible sequelae in future through an outcome tree or disease progression model. |
|  | Life expectancy for YLL | The life table that was used to assess YLL  *Relevant life-tables*: Aspirational standard life tables, i.e., WHO standard life table, GBD standard life table OR National life tables or national life expectancy (i.e., country-specific) |
|  | Disease model | A disease model is a causal chain of a disease that describes health states and their transition probabilities over time. *Did the authors report the disease model that they have used to assess BoD?* [Yes/No] |
|  | Injury classification approach | Cause-of-injury category [Yes/No]; Cause-of-nature category [Yes/No]  Was a cause to nature of injury matrix used for the YLL, YLD, or DALY calculation? [Yes/No] |
|  | Injuries with lifelong consequences | Did the authors mention how the proportions of incident cases in each of the cause-of-injury categories that resulted in each of the cause-of-nature-injury categories were estimated? [Yes/No] |
|  | DW: source | The source of the set(s) of disability weights (DWs) that were used to assess YLD.  *Relevant sources*: GBD DWs, Dutch DWs, Empirical DWs etc. |
|  | DW: elicitation method  (only if study developed own DWs) | Methods for eliciting health state valuations  *Relevant methods*: Visual Analogue Scale (VAS), Person Trade-Off (PTO), Time Trade-Off (TTO), etc. |
|  | DW: panel of judges  (only if study developed own DWs) | The panel of judges whose preferences were obtained to assess DWs  *Relevant panel composition*: medical experts, healthcare professionals, policymakers, patients or people with disabilities, patients’ families, etc. |
|  | DW: severity distribution | The proportion of cases with e.g., mild, moderate or severe health state^1^ of a specific outcome for which separate DWs are available.  *Was a severity distribution used/reported by the authors?* [Yes/No]; [Global/National]  ^1^: a health state reflects a combination of signs or symptoms that result in a certain amount of health loss |
|  | Comorbidity adjustment (YLD calculation) | Adjustment of YLD data for comorbidity^2^ [Yes/No]  ^2^: multiple conditions co-existing in one individual |
|  | Methods co-morbidity adjustment (YLD calculation) | - Approach(es) used to deal with the impact of comorbidity in a BoD study e.g., Standard simulation method, etc  - Approach(es) that can be used to adjust DW’ data for comorbidity  e.g., Additive approach, Multiplicative approach, Maximum limit approach etc |
|  | Social weighting:  age weighting | By incorporating age-weighting into DALY implies that the value of life depends on age; a lower weight of healthy life years lived is given at younger and at older ages – known as ‘non-uniform DALY’ [Yes/No] |
|  | Social weighting:  time discounting | Time-discounting discounts future years of healthy life lived using a rate of 3% or an alternative set of 0% [Yes/No] |
|  | Social weighting: discounting rate | ‘*uniform DALY’*; age-weighting and 3% time-discounting rate; ‘*non-uniform DALY’*; no age-weighting, no time-discounting rate; ‘*age-weighting DALY’*; age-weighting, no time-discounting rate; ‘*time-discounting DALY’*; no age-weighting, 3% time-discounting rate [%] |
| Uncertainty | Uncertainty analysis | An estimation of range or distribution of uncertainty in estimates based on an assessment of the uncertainty or confidence intervals for all data and parameter inputs [Yes/No] |
|  | Uncertainty analysis: method | *Relevant methods of uncertainty in DALY calculations:* Parameter uncertainty, Structural or model uncertainty, Methodological uncertainty |
|  | Sensitivity analysis | Analysis of how the impact of uncertainties of one or more input variables can lead to uncertainties in data inputs or assumptions [Yes/No] |
|  | Scenario analysis | The current or future disease burden is compared with the BoD if one element is changed (e.g., life expectancy, DW or severity distribution) [Yes/No] |
|  | Scenario analysis: element changed | Element that was changed for the scenario analysis (e.g., life expectancy, DW or severity distribution) |
| BoD: burden of disease; DALY: disability adjusted life years; DW: disability weight; GBD: Global Burden of Disease; PMID: PubMed Identifier; YLD: years lost due to disability; YLL: years of life lost due to premature mortality; WHO: World Health Organisation | | |

**Chapter 2**

**Systematic review analysis of all-cause BoD studies**

***Study types per study classification and geographic location***

Figure 1 illustrates the number of GBD-linked and independent all-cause BoD studies per multi-country and single-country category. As can be seen in Table 1, 77 out of the 125 BoD studies (63%) performed an all-cause BoD assessment examining various Group I, Group II, and Group III causes of disease. Out of these 77 studies, 24 (32%) were multi-country GBD-linked studies. Among the all-cause single-country studies, 32 (60%) were independent studies, whereas the remaining 21 (40%) were GBD-linked studies. The number of published all-cause independent BoD studies varied by country, with the lowest number in Russia (n=1), Sweden (n=1), and Switzerland (n=1), and the highest number in Spain (n=6), followed by Estonia (n=5), and Scotland (n=4). The highest number of single-country GBD-linked studies was seen in Norway (n=4 out of 21).

**Figure 1:** Number of GBD-linked and independent all-cause burden of disease studies per multi-country and single-country category

**Table 1:** Number of GBD-linked and independent single-country and multi-country all-cause BoD studies

|  | ***All-cause BoD studies (n=77)*** | |
| --- | --- | --- |
|  | GBD-linked BoD assessments | Independent BoD assessments |
| Single-country | n=21 (47%) | n=32 (100%) |
| Multi-country | n=24 (53%) | n=0 (0%) |

**YLD methodological choices in all-cause burden of disease studies**

***Prevalence-based versus Incidence-based calculations***

Across the all-cause single-country independent studies, 24 out of 32 included own YLD estimates; 13 studies followed the prevalence-based approach, nine the incidence-based approach, and two did not report the approach used to assess YLDs. All all-cause single-country studies quantifying YLDs from the incidence-based approach were performed before 2010 (e.g., Spain, Turkey, Serbia etc). In contrast, some national BoD studies conducted before 2010 (e.g., the Netherlands, Estonia) applied the prevalence approach to assess YLDs. All-cause BoD studies undertaken in Scotland, Germany, and the Netherlands have calculated prevalence-based YLDs. Notably, after 2010, more and more countries (e.g., Albania, Belgium, Greece, Norway, Poland, Russia, Spain, Sweden, the Ukraine etc) quantified disease burden for a variety of causes of ill-health outcome using the prevalence-based GBD YLD estimates (see Figure 2; appendix file, p.14).

***Use of disability weights***

Two all-cause single-country GBD-linked BoD studies (5%) did not report on the DWs used. More than half of independent all-cause BoD studies (58%) used the GBD DWs. Five (21%) and three (13%) independent BoD studies applied DWs developed by the Estonian and Dutch studies, respectively. One single-country independent study (4%) combined the GBD DWs with the Dutch DWs and one BoD study (4%) derived YLDs by applied the ratio of YLD to YLL.

**YLL methodological choices in all-cause burden of disease studies**

***Choice of life-table***

Among the single-country independent studies, 17 used aspirational life tables (n=17 out of 32), such as those developed by the GBD study. The remaining 15 BoD studies (n=15 out of 32) used country-specific life tables or life expectancies. These single-country assessments were carried out in the Netherlands, Germany, Estonia, and Scotland.

**Figure 2:** Number of all-cause single-country burden of disease studies (N=53) by type of analysis, 1990-2019

**Chapter 3**

**Reference list of the included injury-specific and all-cause disease burden studies (N=125)**

| Author(s) | Year | Title | Country(s) / Region | Study type |
| --- | --- | --- | --- | --- |
| Agardh et al | 2018 | Hälsoutvecklingen i Stockholm bättre än i övriga landet - Jämförelse av hälsoläget utifrån globala Sjukdomsbördeprojektet | Sweden | All-cause BoD study |
| Akgün et al | 2007 | Estimating mortality and causes of death in Turkey: methods, results and policy implications | Turkey | All-cause BoD study |
| Aldridge et al | 2017 | Injuries: a call for public health action in Europe; An update using the 2015 global health estimates | WHO European Region | Injury-specific study |
| Bikbov et al | 2014 | Mortality landscape in the Global Burden of Diseases, Injuries and Risk Factors Study | Global | All-cause BoD study |
| Begg & Niels | 2006 | Global burden of injury in the year 2000: an overview of methods | Global | Injury-specific study |
| Bowie et al | 1997 | Estimating the burden of disease in an English region | South and West Region of England | All-cause BoD study |
| Burazeri et al | 2014 | National Health Report: Health Status of the Albanian Population | Albania | All-cause BoD study |
| Catala-Lopez et al | 2013 | Burden of disease assessment with summary measures of population health for the Region of Valencia, Spain: a population-based study | Spain (Region of Valencia) | All-cause BoD study |
| Catala-Lopez et al | 2013 | Carga de enfermedad en adolescentes y jóvenes en España | Spain | All-cause BoD study |
| Cieza et al | 2021 | Global estimates of the need for rehabilitation based on the Global Burden of Disease study 2019: a systematic analysis for the Global Burden of Disease Study 2019 | Global | All-cause BoD study |
| Cortes Garcia et al | 2004 | Carga de enfermedad de la población española menor de 15 años para el año 1999 | Spain | All-cause BoD study |
| Cuadras & Ricart | 2014 | Carga de enfermedad en la comunidad autónoma de Cataluña utilizando años de vida ajustados por discapacidad (2005-2010) | Spain; Catalunya region (Tarragones subregion) | All-cause BoD study |
| Crowe et al | 2020 | Global trends of hand and wrist trauma: a systematic analysis of fracture and digit amputation using the Global Burden of Disease 2017 Study | Global | Injury-specific study |
| Direção-Geral da Saúde | 2018 | Portugal: The Nation's Health 1990-2016; an overview of the Global Burden of Disease Study 2016 Results | Portugal | All-cause BoD study |
| Dodhia & Phillips | 2008 | Measuring burden of disease in two inner London boroughs using Disability Adjusted Life Years | London (boroughs of Lambeth and Southwark) | All-cause BoD study |
| Dhondt et al | 2012 | Translating road safety into health outcomes using a quantitative impact assessment model | Flanders & Brussels | Injury-specific study |
| Dhondt et al | 2013 | Health burden of road traffic accidents, an analysis of clinical data on disability and mortality exposure rates in Flanders and Brussels | Flanders & Brussels | Injury-specific study |
| Fattahov & Piankova | 2018 | The Years of Life Lost as a Result of Road Traffic Accidents In Russia in 2000-2012 | Russia | Injury-specific study |
| Franklin et al | 2020 | The burden of unintentional drowning: global, regional and national estimates of mortality from the Global Burden of Disease 2017 Study | Global | Injury-specific study |
| Ganczakl et al | 2020 | Changes in disease burden in Poland between 1990-2017 in comparison with other Central European countries: A systematic analysis for the Global Burden of Disease Study 2017 | Poland | All-cause BoD study |
| Genova-Maleras et al | 2011 | Burden of disease in the elderly population in Spain | Spain | All-cause BoD study |
| Genova-Maleras et al | 2012 | Measuring the burden of disease and injury in Spain using disability-adjusted life years: an updated and policy-oriented overview | Spain | All-cause BoD study |
| Gobbino et al | 2012 | Relazione sullo stato dell’incidentalità in Friuli Venezia Giulia | Italy (Friuli Venezia Giulia) | Injury-specific study |
| Gore et al | 2011 | Global burden of disease in young people aged 10-24 years: a systematic analysis | Global | All-cause BoD study |
| Grant et al | 2015 | The Scottish Burden of Disease (SBoD) study; Age–gender report | Scotland | All-cause BoD study |
| Grant et al | 2017 | The burden of disease is generally greater in more deprived areas, and that burden is more likely to be fatal | Scotland | All-cause BoD study |
| Haagsma et al | 2008 | Novel empirical disability weights to assess the burden of non-fatal injury | Netherlands | Injury-specific study |
| Haagsma et al | 2012 | Improved and standardized method for assessing years lived with disability after injury | Netherlands, Ceres, Thailand | Injury-specific study |
| Haagsma et al | 2016 | The global burden of injury: incidence, mortality, disability-adjusted life years and time trends from the Global Burden of Disease study 2013 | Global | Injury-specific study |
| Haagsma et al | 2020 | Falls in older aged adults in 22 European countries: incidence, mortality and burden of disease from 1990 to 2017 | GBD Western Europe | Injury-specific study |
| Haagsma et al | 2020 | Burden of injury along the development spectrum: associations between the Socio-demographic Index and disability-adjusted life year estimates from the Global Burden of Disease Study 2017 | Global | Injury-specific study |
| Hagen et al | 2020 | Estimating the future burden of hip fractures in Norway. A NOREPOS study | Norway | Injury-specific study |
| Haro et al | 2014 | The burden of disease in Spain: results from the global burden of disease study 2010 | Spain | All-cause BoD study |
| Hay et al | 2018 | Global, regional, and national disability-adjusted life-years (DALYs) for 333 diseases and injuries and healthy life expectancy (HALE) for 195 countries and territories, 1990–2016: a systematic analysis for the Global Burden of Disease Study 2016 | Global | All-cause BoD study |
| Hoeymans et al | 2010 | De ziektelast van suïcide en suïcidepogingen | Netherlands | Injury-specific study |
| Holtslag et al | 2008 | Individual and population burdens of major trauma in the Netherlands | Netherlands | Injury-specific study |
| Hilderink et al | 2020 | Dutch DALYs, current and future burden of disease in the Netherlands | Netherlands | All-cause BoD study |
| James et al | 2018 | Global, regional, and national incidence, prevalence, and years lived with disability for 354 diseases and injuries for 195 countries and territories, 1990–2017: a systematic analysis for the Global Burden of Disease Study 2017 | Global | All-cause BoD study |
| James et al | 2019 | Epidemiology of injuries from fire, heat and hot substances: global, regional and national morbidity and mortality estimates from the Global Burden of Disease 2017 study | Global | Injury-specific study |
| James et al | 2019 | Global, regional, and national burden of traumatic brain injury and spinal cord injury, 1990–2016: a systematic analysis for the Global Burden of Disease Study 2016 | Global | Injury-specific study |
| James et al | 2020 | Global injury morbidity and mortality from 1990 to 2017: results from the Global Burden of Disease Study 2017 | Global | Injury-specific study |
| Jankovic et al | 2007 | The burden of disease and injury in Serbia | Serbia, Serbia and Montenegro | All-cause BoD study |
| Johnell & Kanis | 2004 | An estimate of the worldwide prevalence, mortality and disability associated with hip fracture | World Bank regions | Injury-specific study |
| Karelson | 2016 | Eesti rahvastiku tervisekaotus 2013. Aastal | Estonia | All-cause BoD study |
| Kassebaum et al | 2016 | Global, regional, and national disability-adjusted life-years (DALYs) for 315 diseases and injuries and healthy life expectancy (HALE), 1990-2015: a systematic analysis for the Global Burden of Disease Study 2015 | Global | All-cause BoD study |
| Kissimova-Skarbek | 2016 | Approaches to Disease Burden Measurement: Disability-Adjusted Life Years (DALYs) Globally and in Poland, and National Income Lost Due to Disease in Poland, 1990–2015 | Poland | All-cause BoD study |
| Khan et al | 2020 | Global Trends and Forecast of the Burden of Adverse Effects of Medical Treatment: Epidemiological Analysis Based on the Global Burden of Disease Study | WHO regions (Africa, the Americas, Southeast Asia, Europe, Eastern Mediterranean region, and Western Pacific region) | Injury-specific study |
| Knudsen et al | 2016 | Sykdomsbyrde i Norge 1990-2013 | Norway | All-cause BoD study |
| Knudsen et al | 2017 | Sykdomsbyrde i Norge 2015; Resultater fra Global Burden of Diseases, Injuries, and Risk Factors Study 2015 (GBD 2015) | Norway | All-cause BoD study |
| Knudsen et al | 2019 | Life expectancy and disease burden in the Nordic countries: results from the Global Burden of Diseases, Injuries, and Risk Factors Study 2017 | Nordic countries (Denmark, Finland, Iceland, Norway, Sweden), Greenland | All-cause BoD study |
| Kobyakova et al | 2014 | Динамика числа потерянных лет жизни (DALY) в результате преждевременной смертности детей в возрасте 0–17 лет в Томской области в 2008–2012 гг (Dynamics of Disability Adjusted Life Years (DALY) as a Result of Premature Mortality of Children at the Age of 0–17 Years in Tomsk Region in 2008–2012) | Tomsk Region of Russian | All-cause BoD study |
| Kyu et al | 2018 | Global, regional, and national disability-adjusted life-years (DALYs) for 359 diseases and injuries and healthy life expectancy (HALE) for 195 countries and territories, 1990-2017: a systematic analysis for the Global Burden of Disease Study 2017 | Global | All-cause BoD study |
| Lai et al | 2004 | Haiguskoormuse tõttu kaotatud eluaastad Eestis: seosed riskifaktoritega ja riskide vähendamise kulutõhusus | Estonia | All-cause BoD study |
| Lai et al | 2006 | Maakondlik haiguskoormus Eestis 2000 – 2004 | Estonia | All-cause BoD study |
| Lai & Kohler | 2009 | Burden of Disease of Estonian population | Estonia (Hiiu, Saare, Rapla, Ida-Viru, Põlva, Võru, Harju, Tartu, Lääne, Pärnu, Viljandi, Järva, Lääne-Viru, Jõgeva, Valga) | All-cause BoD study |
| Lai et al | 2009 | Measuring burden of disease in Estonia to support public health policy | Estonia | All-cause BoD study |
| Lalloo et al | 2020 | Epidemiology of facial fractures: incidence, prevalence and years lived with disability estimates from the Global Burden of Disease 2017 study | Global | Injury-specific study |
| Lapostolle et al | 2007 | Sensitivity analysis in summary measure of population health in France | France | All-cause BoD study |
| Lapostolle et al | 2009 | The burden of road traffic accidents in a French Department: The description of the injuries and recent changes | France | Injury-specific study |
| Lekhan & Kriachkova | 2019 | The system of measures to improve the health of the population of Ukraine based on the analysis of the global burden of diseases and its risk factors | Ukraine | All-cause BoD study |
| Leilveld et al | 2020 | Epidemiologic trends for isolated tibia shaft fracture admissions in The Netherlands between 1991 and 2012 | Netherlands | Injury-specific study |
| Lin et al | 2016 | The global distribution of the burden of road traffic injuries: Evolution and intra-distribution mobility | Global | Injury-specific study |
| Ljung et al | 2005 | Socioeconomic differences in the burden of disease in Sweden | Sweden | All-cause BoD study |
| Lopez et al | 2006 | Global and regional burden of disease and risk factors, 2001: systematic analysis of population health data | Global | All-cause BoD study |
| Lozano et al | 2012 | Global and regional mortality from 235 causes of death for 20 age groups in 1990 and 2010: a systematic analysis for the Global Burden of Disease Study 2010 | Global | All-cause BoD study |
| Lukaschek et al | 2012 | Suicide mortality in comparison to traffic accidents and homicides as causes of unnatural death. an analysis of 14,441 cases in Germany in the year 2010 | Germany | Injury-specific study |
| Lunevicius & Haagsma | 2018 | An analogy between socioeconomic deprivation level and loss of health from adverse effects of medical treatment in England | Nine English regions | Injury-specific study |
| Lyons et al | 2017 | Disability Adjusted Life Year (DALY) estimates for injury utilising the European Injury Data Base (IDB) | EU-28 | Injury-specific study |
| Machado et al | 2011 | Carga Global da Doença na região Norte de Portugal | Northern region of Portugal | All-cause BoD study |
| Maertens de Noordhout et al | 2018 | Changes in health in Belgium, 1990–2016: a benchmarking analysis based on the global burden of disease 2016 study | Belgium | All-cause BoD study |
| Mariotti et al | 2003 | Years of life lost due to premature mortality in Italy | Italy | All-cause BoD study |
| Majdan et al | 2017 | Years of life lost due to traumatic brain injury in Europe: A cross-sectional analysis of 16 countries | EU-16 | Injury-specific study |
| Melse & Kramers | 1998 | Berekeningen van de ziektelast in Nederland. Achtergronddocument bij VTV-1997 deel III, hoofdstuk 7 | Netherlands | All-cause BoD study |
| Melse et al | 2000 | A national burden of disease calculation: Dutch disability-adjusted life-years. Dutch Burden of Disease Group | Netherlands | All-cause BoD study |
| Mesalles-Naranjo  et al | 2018 | Trends and inequalities in the burden of mortality in Scotland 2000–2015 | Scotland | All-cause BoD study |
| Mokdad et al | 2016 | Global burden of diseases, injuries, and risk factors for young people's health during 1990-2013: a systematic analysis for the Global Burden of Disease Study 2013 | Global | All-cause BoD study |
| Monasta et al | 2019 | Italy’s health performance, 1990–2017: findings from the Global Burden of Disease Study 2017 | Italy | All-cause BoD study |
| Murray et al | 2001 | The Global Burden of Disease 2000 project: aims, methods and data sources | Global | All-cause BoD study |
| Murray et al | 2012 | Disability-adjusted life years (DALYs) for 291 diseases and injuries in 21 regions, 1990-2010: a systematic analysis for the Global Burden of Disease Study 2010 | Global | All-cause BoD study |
| Murray et al | 2013 | UK health performance: findings of the Global Burden of Disease Study 2010 | Global | All-cause BoD study |
| Murray et al | 2015 | Global, regional, and national disability-adjusted life years (DALYs) for 306 diseases and injuries and healthy life expectancy (HALE) for 188 countries, 1990-2013: quantifying the epidemiological transition | Global | All-cause BoD study |
| Naghavi et al | 2019 | Global, regional, and national burden of suicide mortality 1990 to 2016: Systematic analysis for the Global Burden of Disease Study 2016 | Global | Injury-specific study |
| Naghavi et al | 2017 | Global, regional, and national age-sex specific mortality for 264 causes of death, 1980–2016: a systematic analysis for the Global Burden of Disease Study 2016 | Global | All-cause BoD study |
| Newton et al | 2015 | Changes in health in England, with analysis by English regions and areas of deprivation, 1990–2013: a systematic analysis for the Global Burden of Disease Study 2013 | England | All-cause BoD study |
| Nielsen et al | 2004 | Burden of mortality in Greenland--today and tomorrow | Greenland | All-cause BoD study |
| Øverland et al | 2018 | Sykdomsbyrden i Norge 2016; Resultater fra Global Burden of Diseases, Injuries, and Risk Factors Study 2016 (GBD 2016) | Norway | All-cause BoD study |
| Peden et al | 2002 | The injury chart book: A graphical overview of the global burden of injuries | Global | Injury-specific study |
| Plass et al | 2014 | Trends in disease burden in Germany: results, implications and limitations of the Global Burden of Disease study | Germany | All-cause BoD study |
| Polinder et al | 2007 | Assessing the burden of injury in six European countries | Austria, Denmark, Ireland, Netherlands, Norway, and United Kingdom (England and Wales) | Injury-specific study |
| Polinder et al | 2010 | Burden of injury in childhood and adolescence in 8 European countries | Austria, Denmark, Ireland, Latvia, Netherlands, Norway, Slovenia and the United Kingdom (England, Wales) | Injury-specific study |
| Polinder et al | 2012 | Epidemiological burden of minor, major and fatal trauma in a national injury pyramid | Netherlands | Injury-specific study |
| Polinder et al | 2015 | Burden of road traffic injuries: Disability-adjusted life years in relation to hospitalization and the maximum abbreviated injury scale | Netherlands | Injury-specific study |
| Prins et al | 2021 | Trends in incidence rate, health care use, and costs due to rib fractures in the Netherlands | Netherlands | Injury-specific study |
| Reiner et al | 2019 | Diseases, Injuries, and Risk Factors in Child and Adolescent Health, 1990 to 2017: Findings From the Global Burden of Diseases, Injuries, and Risk Factors 2017 Study | Global | All-cause BoD study |
| Rommel et al | 2018 | BURDEN 2020 - Burden of disease in Germany at the national and regional level | Germany | All-cause BoD study |
| Roth et al | 2018 | Global, regional, and national age-sex-specific mortality for 282 causes of death in 195 countries and territories, 1980–2017: a systematic analysis for the Global Burden of Disease Study 2017 | Global | All-cause BoD study |
| Santos et al | 2019 | The state of health in the European Union (EU-28) in 2017: an analysis of the burden of diseases and injuries | EU-28 countries | All-cause BoD study |
| Santric Milicevic  et al | 2009 | Serbia within the European context: An analysis of premature mortality | Serbia (Kosovo and Metohija were excluded); EURO A; EURO B; EURO C | All-cause BoD study |
| Scholten et al | 2014 | Traumatic brain injury in the Netherlands: Incidence, costs and disability-adjusted life years | Netherlands | Injury-specific study |
| Schopper et al | 2000 | Estimating the burden of disease in one Swiss canton: what do disability adjusted life years (DALY) tell us? | Swiss canton; Geneva | All-cause BoD study |
| Snijders et al | 2016 | Ziektelast en kosten van letsel door geweld | Netherlands | Injury-specific study |
| Sethi et al | 2008 | European Report on Child Injury Prevention | WHO European Region | Injury-specific study |
| Soriano et al | 2018 | The burden of disease in Spain: Results from the Global Burden of Disease 2016 | Spain | All-cause BoD study |
| Starodubov et al | 2018 | The burden of disease in Russia from 1980 to 2016: a systematic analysis for the Global Burden of Disease Study 2016 | Russia Federation | All-cause BoD study |
| Steel et al | 2018 | Changes in health in the countries of the UK and 150 English Local Authority areas 1990-2016: a systematic analysis for the Global Burden of Disease Study 2016 | UK; England; Scotland; Wales; Northern Ireland; 150 English Upper-Tier Authorities | All-cause BoD study |
| Spronk et al | 2020 | Improved and standardized method for assessing years lived with disability after burns and its application to estimate the non-fatal burden of disease of burn injuries in Australia, New Zealand and the Netherlands | Netherlands, New Zealand, and Australia | Injury-specific study |
| Stockton et al | 2016 | The Scottish Burden of Disease (SBoD) study; Deprivation report | Scotland | All-cause BoD study |
| NHS Health Scotland | 2016 | The Scottish Burden of Disease (SBoD) study; Suicide and self-harm technical overview | Scotland | Injury-specific study |
| NHS Health Scotland | 2016 | The Scottish Burden of Disease (SBoD) study; Falls technical overview | Scotland | Injury-specific study |
| Tainio | 2015 | Burden of disease caused by local transport in Warsaw, Poland | Warsaw; Poland | Injury-specific study |
| Tainio et al | 2014 | Severity of injuries in different modes of transport, expressed with disability-adjusted life years (DALYs) | Sweden | Injury-specific study |
| Tollanes et al | 2018 | Disease burden in Norway in 2016 | Norway | All-cause BoD study |
| Twisk et al | 2017 | Road injuries, health burden, but not fatalities make 12- to 17-year olds a high risk group in the Netherlands | Netherlands | Injury-specific study |
| Tyrovolas et al | 2018 | The burden of disease in Greece, health loss, risk factors, and health financing, 2000–16: an analysis of the Global Burden of Disease Study 2016 | Greece | All-cause BoD study |
| Ünüvar et al | 2006 | Turkey Burden of Disease Study | Turkey | All-cause BoD study |
| Valent & Zanier | 2015 | A population-based study of the years of life lost in the Friuli Venezia Giulia region, Italy | Italy (Friuli Venezia Giulia) | All-cause BoD study |
| Valent et al | 2007 | Burden of disease attributable to selected environmental factors and injury among children and adolescents in Europe | WHO European Region | Injury-specific study |
| Vlajinac et al | 2008 | Years of life lost due to premature death in Serbia (excluding Kosovo and Metohija) | Serbia (excluding Kosovo and Metohija) | All-cause BoD study |
| Vos et al | 2015 | Global, regional, and national incidence, prevalence, and years lived with disability for 301 acute and chronic diseases and injuries in 188 countries, 1990-2013: a systematic analysis for the Global Burden of Disease Study 2013 | Global | All-cause BoD study |
| Vos et al | 2012 | Years lived with disability (YLDs) for 1160 sequelae of 289 diseases and injuries 1990–2010: a systematic analysis for the Global Burden of Disease Study 2010 | Global | All-cause BoD study |
| Vos et al | 2020 | Global burden of 369 diseases and injuries in 204 countries and territories, 1990–2019: a systematic analysis for the Global Burden of Disease Study 2019 | Global | All-cause BoD study |
| Wengler et al | 2021 | Years of Life Lost to Death: A comprehensive analysis of mortality in Germany conducted as part of the BURDEN 2020 project | Germany | All-cause BoD study |
| Weijermars et al | 2018 | Burden of injury of serious road injuries in six EU countries | Austria, Belgium, England, Spain, Netherlands, France (Rhône region) | Injury-specific study |
| Weijermars et al | 2016 | Health burden of serious road injuries in the Netherlands | Netherlands | Injury-specific study |
| WHO | 2008 | The global burden of disease: 2004 update | Global | All-cause BoD study |
